# Supplementary material for: Expression of IFN-Gamma is significantly reduced during severity of covid-19 infection in hospitalized patients
Source: PLoS One. 2023 Sep 27;18(9):e0291332. doi: 10.1371/journal.pone.0291332 (PMC10530045; doi:10.1371/journal.pone.0291332)
Supplement: Attachment — Submitted filename: 2nd Rebuttal - 2nd Response to Reviewers.docx [file pone.0291332.s002.docx]

Dr. Bashir Uddin,

Academic Editor,

PLOS ONE,	Dated: 15-08-2023

**Subject: Rebuttal; Response to Reviewers**

Dear Sir,

We are extremely pleased regarding the review that only a minor revision remains. The 2^nd^ comment by the esteemed reviewer 2, as pointed out by the respected academic editor along with an appropriate response is as following:

Comment # 2 by Reviewer # 2:

The amount of IL-7 expression in figure 3 is not particularly significant, which might be an artifact. However, the evidence should be validated by reproducible cell-based or mouse models.

Response:

The title has been changed and now the main emphasis is on interferon gamma instead of IL-7. Similar changes have been made in the main body of the text.

However, as for the second part of the comment by reviewer 2, any further validation about whether the IL-7 expression is actual or just an artifact through reproducible cell-based or mouse models, warrants a separate study. We agree with the esteemed reviewer that it might just be an artifact, nonetheless, we report it as per the data collected. Moreover, we try to make no extended claims based on this and have corrected anything as such within the version of the manuscript submitted.

We sincerely hope that this clarifies any ambiguities and makes the manuscript appropriate for acceptance.

Thank you.

Please do extent any queries. Take care.

Best regards

Dr. Amjad Khan

Corresponding author

Email: [amjadkhan@qau.edu.pk](mailto:amjadkhan@qau.edu.pk)
